# Are Suicide Rates Related to the Psychiatrist Density? A Cross-National Study

**DOI:** 10.3389/fpubh.2015.00280

**Published:** 2016-01-06

**Authors:** Leo Sher

**Affiliations:** ^1^James J. Peters Veterans’ Administration Medical Center and Icahn School of Medicine at Mount Sinai, New York, NY, USA

**Keywords:** psychiatrist density, gross national income, suicide, European Union, public health

## Abstract

**Introduction:**

Most suicide victims have a diagnosable psychiatric disorder. Treatment of psychiatric disorders should reduce the number of suicides. Higher psychiatrist-per-­population ratio increases the opportunity for contact between the patient and psychiatrist. It is reasonable to hypothesize that the higher psychiatrist density (PD) is associated with lower suicide rates. The aim of this study is to examine the association between suicide rates and the PD in the European Union countries. These countries are economically and culturally connected and located on the same continent. This is an attempt to study a relatively homogenous sample.

**Methods:**

Correlations were computed to examine relationships between age-­standardized suicide rates in women and men, the PD, and the gross national income (GNI) per capita. Partial correlations were used to examine the relation between the PD and age-standardized suicide rates in women and men controlling for the GNI per capita.

**Results:**

Higher suicide rates in women correlated with the higher PD. Controlling for the GNI per capita, the PD positively correlated with suicide rates both in women and in men. There was a trend toward a negative correlation between the GNI per capita and suicide rates in men. The PD was positively associated with the GNI per capita.

**Conclusion:**

Probably, higher suicide rates directly and/or indirectly affect the decisions made by policy- and lawmakers regarding mental health services and how many psychiatrists need to be trained. The results of this study should be treated with caution because many confounding variables are not taken into account.

## Introduction

Suicidal behavior is a major public health issue and a global phenomenon (1–3). Suicide is complex human behavior with multiple causes that include biological and psychosocial components. The World Health Organization (WHO) reports that every year more than 800,000 people take their own life around the world (1). The WHO recognizes suicide as a public health priority. Suicide is an important public health problem in the U.S. In 2013, there were 41,143 suicides in the U.S., which equates to approximately 112 deaths a day (2). Suicide occurs regardless of race, income, or gender. Men die by suicide at a rate four times higher than women. Reductions in suicide burden require multiple actions, including the development of new research programs and new approaches to suicide research and prevention.

More than 90% of suicide victims have a diagnosable psychiatric disorder (3–8). The most common psychiatric conditions associated with suicide are mood disorders (3–5). It has been reported that 59–87% of suicide victims suffer from depression at the time of suicide (4). Personality disorders, alcohol and substance abuse, anxiety disorders, and schizophrenia are also frequently associated with suicide (5–9). Therefore, treatment of psychiatric disorders should reduce the number of suicides.

Higher psychiatrist-per-population ratio improves access, decreases waiting times, and increases the opportunity for contact between the patient and psychiatrist. Generally, an increase in the physician density tends to lead to physician-induced demand (10). People living in countries with greater psychiatrist density (PD) had a higher probability of seeing a psychiatrist.

The literature suggests that availability of good healthcare services may reduce suicide rates by allowing timely identification and appropriate treatment of psychiatric disorders, providing prompt and effective medical help including resuscitation to those who attempt suicide, allowing appropriate management of risk factors for suicides, and facilitating the implementation of national policies on mental health and suicide prevention (11).

It is reasonable to hypothesize that the higher PD is associated with lower suicide rates. To test this hypothesis, I performed an ecological cross-sectional study. I examined the association between suicide rates and the PD in the European Union countries. These countries are economically and culturally connected and located on the same continent. This is an attempt to study a relatively homogenous sample. Socioeconomic factors may influence suicide rates (12–15). Therefore, I controlled for the gross national income (GNI) per capita.

## Materials and Methods

Information on age-standardized suicide rates in men and women, the PD, and the GNI per capita in the European Union countries was obtained from the WHO database, “Global Health Observatory Data Repository” (Table 1) (16, 17). The age-standardized suicide rate is a weighted average of the age-specific mortality rates per 100,000 persons, where the weights are the proportions of persons in the corresponding age groups of the WHO standard population (18). The 2012 WHO data on suicide rates were used (16).

**Table 1:** **Age-standardized suicide rates, the psychiatrist density, and the gross national income per capita in the European Union countries**.

Country	Age-standardized suicide rates (per 100,000 people per year)			Psychiatrists working in the mental health sector (per 100,000 people)	Gross national income per capita (GNI; int. $)
	Both sexes	Women	Men		
Austria	11.5	5.4	18.2	19.71	42,990
Belgium	14.2	7.7	21	20.32	39,870
Bulgaria	10.8	5.3	16.6	6.75	15,250
Croatia	11.6	4.5	19.8	10.25	19,700
Cyprus	4.7	1.5	7.7	2.69	29,600
Czech Republic	12.5	3.9	21.5	11.85	24,980
Denmark	8.8	4.1	13.6	9.57	43,200
Estonia	13.6	3.8	24.9	13.87	22,900
Finland	14.8	7.5	22.2	18.37	38,570
France	12.3	6	19.3	14.12	36,690
Germany	9.2	4.1	14.5	15.23	42,860
Greece	3.8	1.3	6.3	14.09	25,680
Hungary	19.1	7.4	32.4	6.52	21,000
Ireland	11	5.2	16.9	6.06	35,090
Italy	4.7	1.9	7.6	10.85	34,070
Latvia	16.2	4.3	30.7	12.05	21,350
Lithuania	28.2	8.4	51	17.76	23,110
Luxembourg	8.7	4.4	13	21.15	59,750
Malta	6	0.7	11.1	3.17	26,940
Netherlands	8.2	4.8	11.7	20.1	42,890
Poland	16.6	3.8	30.5	5.07	21,320
Portugal	8.2	3.5	13.6	4.49	24,750
Romania	10.5	2.9	18.4	5.98	17,300
Slovakia	10.1	2.5	18.5	11.48	24,740
Slovenia	12.4	4.4	20.8	10.21	27,610
Spain	5.1	2.2	8.2	8.08	31,140
Sweden	11.1	6.1	16.2	18.31	43,090
United Kingdom	6.2	2.6	9.8	14.63	34,640

The PD is the number of psychiatrists working in the mental health sector per 100,000 of the population (19). Psychiatrists working in mental health include psychiatrists employed in private and public mental health facilities as well as private practice. The most recent data available in the WHO database were used.

The GNI is the gross national income converted to international dollars using purchasing power parity rates (20). An international dollar has the same purchasing power over the GNI as a U.S. dollar has in the U.S. GNI is the sum of value added by all resident producers plus any product taxes (less subsidies) not included in the valuation of output plus net receipts of primary income (compensation of employees and property income) from abroad. The latest data available in the WHO databank were used.

Correlations were computed to examine relationships between age-standardized suicide rates in women and men, PD, and GNI. Partial correlations were used to examine the relation between PD and age-standardized suicide rates in women and men controlling for GNI.

The following method was used to create scattered plots of partial correlations (21): I created three linear regression models. SNI per capita was an independent variable in every model. In the first, the second, and the third models suicide rates in women, suicide rates in men, and the PD were dependent variables, respectively. I computed the residuals of suicide rates in women, suicide rates in men, and the PD. The partial correlation is equivalent to the correlation between the residuals of the linear regression.

The SPSS program (version 23) was used to perform statistical analysis.

## Results

The WHO have data on all 28 European Union countries included in the study: Austria, Belgium, Bulgaria, Croatia, Cyprus, Czech Republic, Denmark, Estonia, Finland, France, Germany, Greece, Hungary, Ireland, Italy, Latvia, Lithuania, Luxembourg, Malta, Netherlands, Poland, Portugal, Romania, Slovakia, Slovenia, Spain, Sweden, and United Kingdom (Table 1). The mean female and male suicide rates, the PD, and the GNI in the European Union countries were 4.293 ± 1.992 and 18.429 ± 9.413 per 100,000 people per year, 11.883 ± 5.605 per 100,000 people, and $31,110 ± 10,374, respectively.

**Figure 1:**
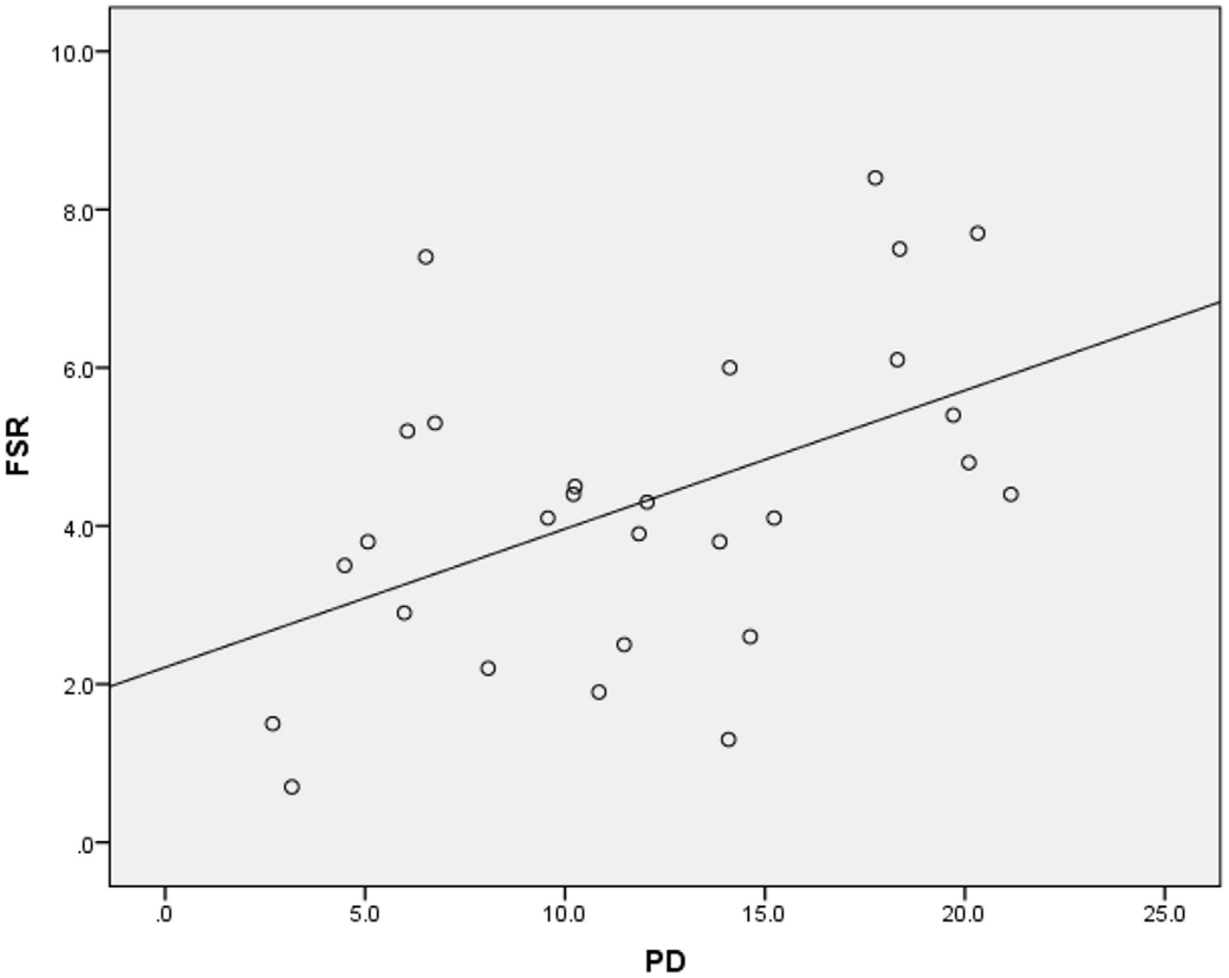
**Correlation between psychiatrist density (PD) and female suicide rates (FSR)**.

**Figure 2:**
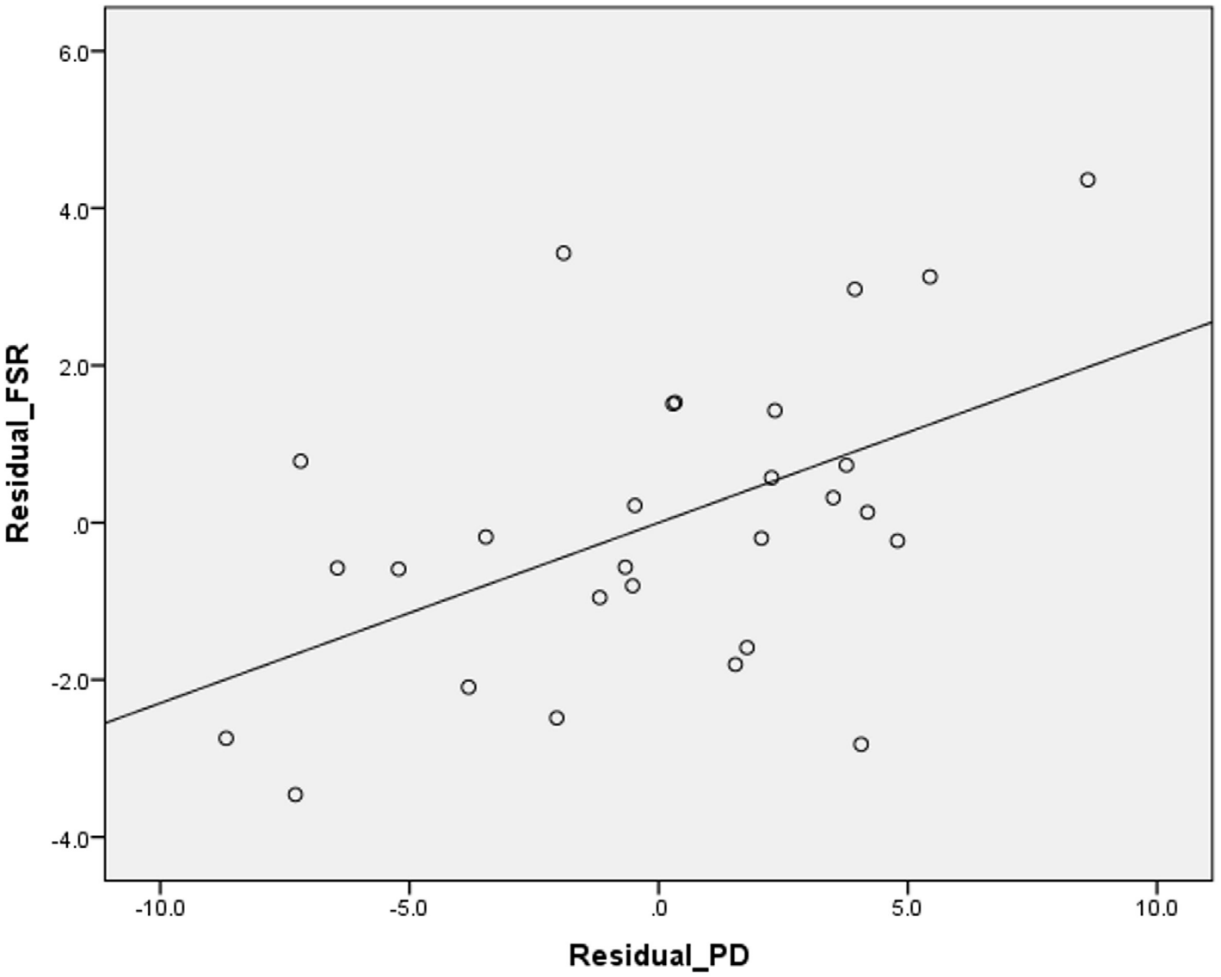
**Correlation between the residuals of the psychiatrist density (PD) and female suicide rates (FSR) (equivalent of partial correlation – please, see Materials and Methods)**.

**Figure 3:**
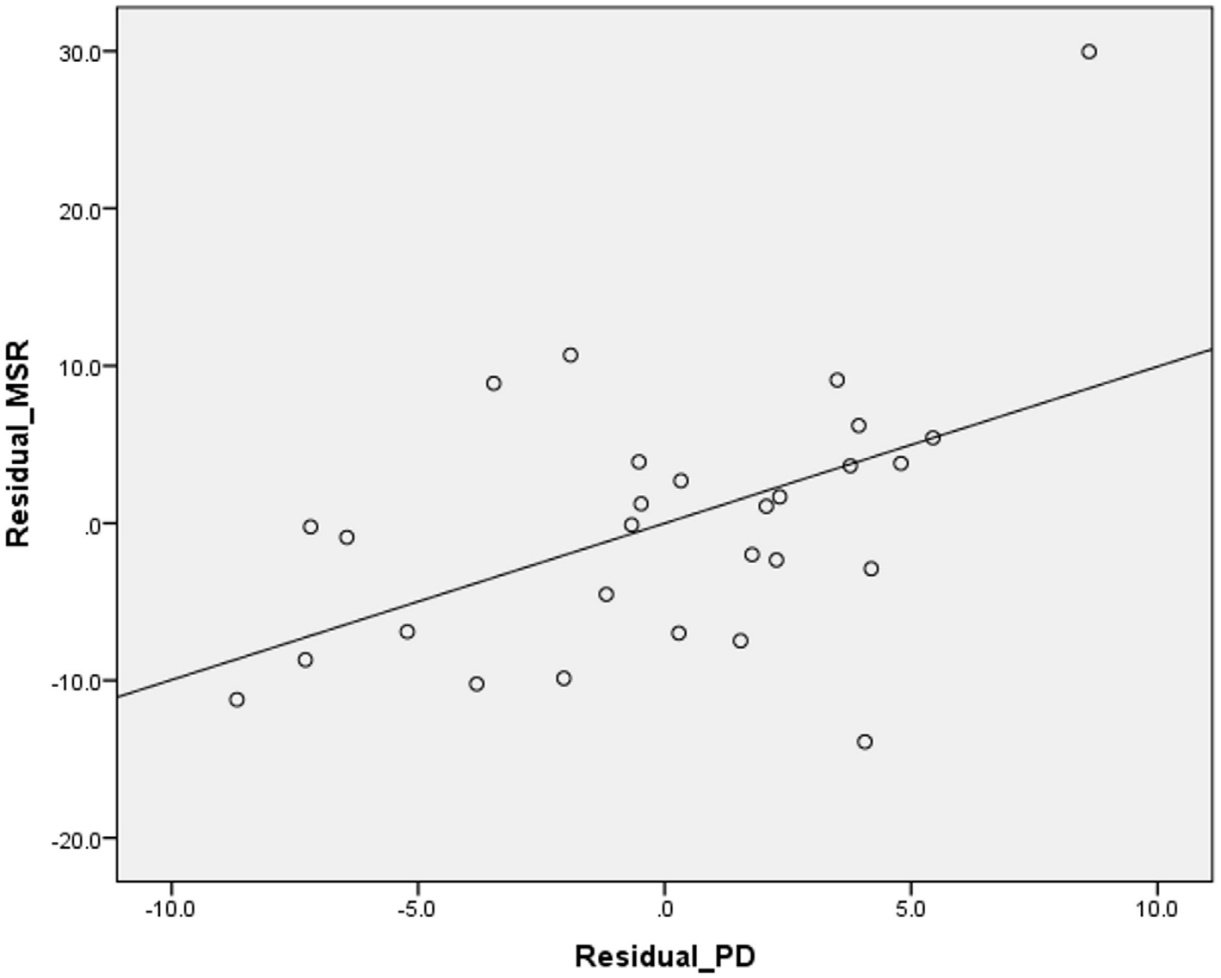
**Correlation between the residuals of the psychiatrist density (PD) and male suicide rates (FSR) (equivalent of partial correlation – please, see Materials and Methods)**.

**Figure 4:**
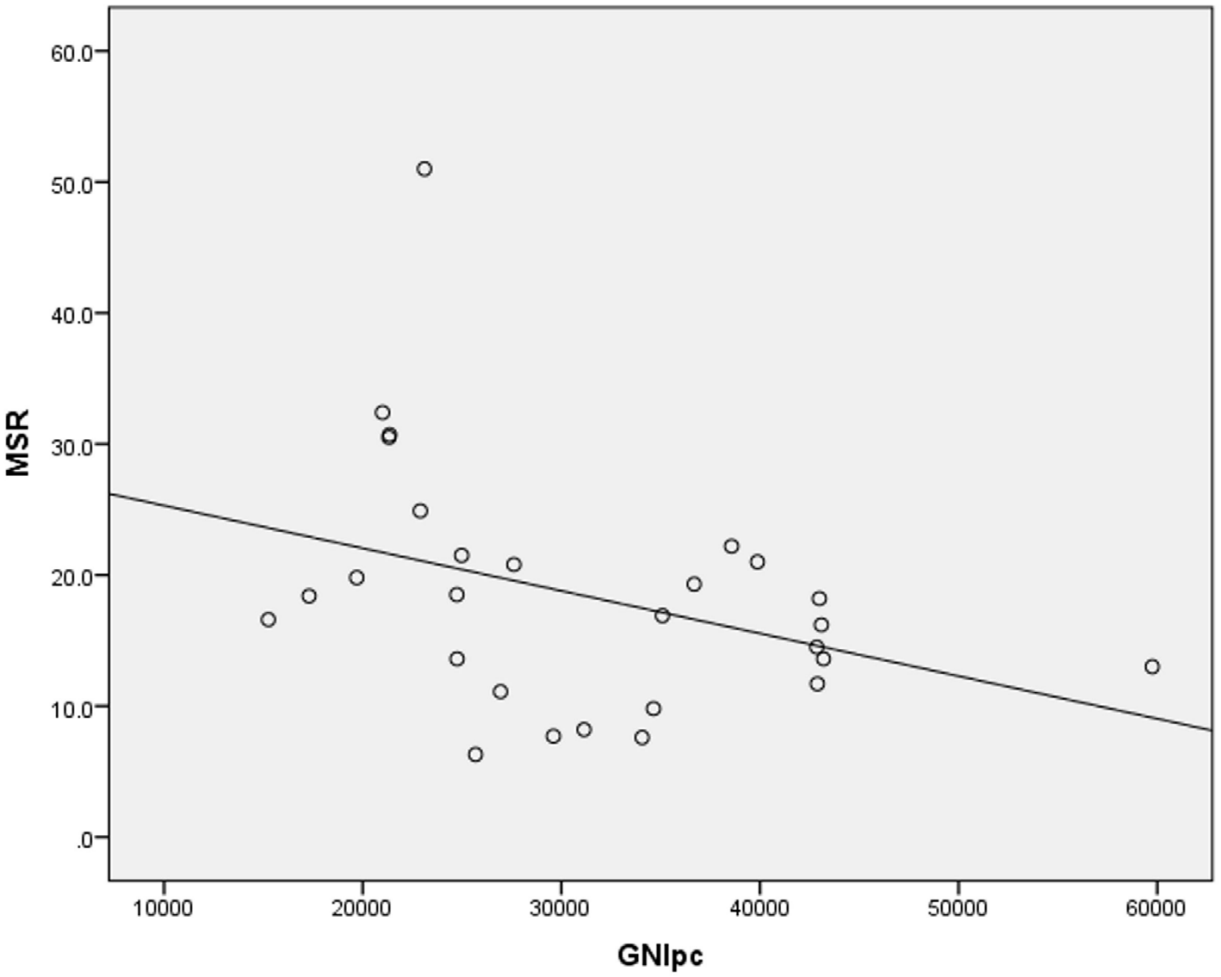
**Correlation between the GNI per capita and male suicide rates**.

**Figure 5:**
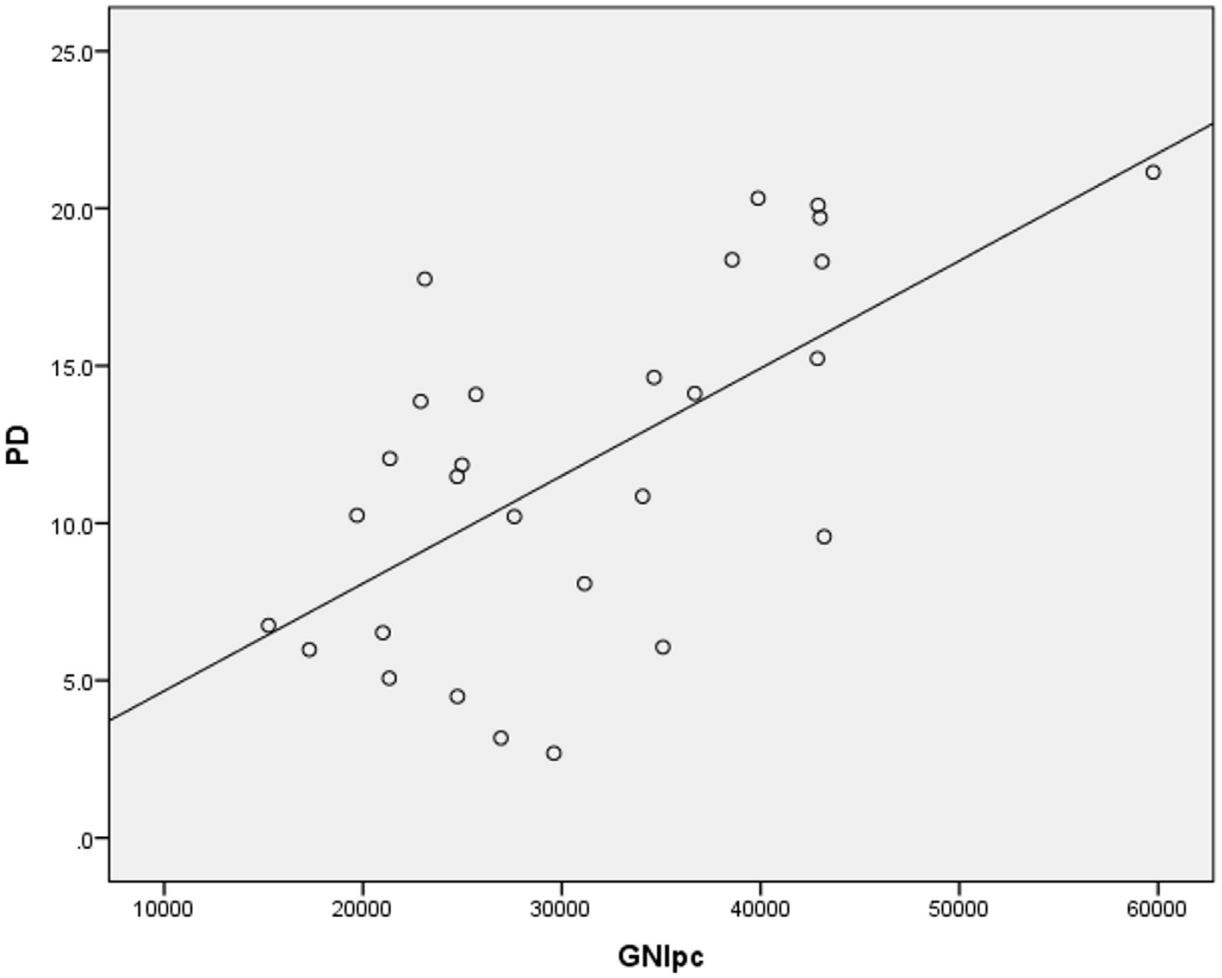
**Correlation between the GNI per capita and the psychiatrist density**.

Higher suicide rates in women correlated with the higher PD (Table 2; Figure 1). Controlling for the GNI per capita, the PD positively correlated with suicide rates both in women and in men (Table 3; Figures 2 and 3). There was a trend toward a negative correlation between the GNI per capita and suicide rates in men (Table 2; Figure 4). The PD was positively associated with the GNI per capita (Table 2; Figure 5).

**Table 2:** **Correlations between age-standardized suicide rates, psychiatrist density, and gross national income per capita**.

		Psychiatrist density	Gross national income per capita
Women	Pearson correlation	0.493	0.166
	Significance (two-tailed)	0.008	0.4
	*N*	28	28
Men	Pearson correlation	0.129	−0.359
	Significance (two-tailed)	0.514	0.61
	*N*	28	28
Psychiatrist density	Pearson correlation		0.633
	Significance (two-tailed)		<0.001
	*N*		28

**Table 3:** **Correlations between age-standardized suicide rates and psychiatrist density controlling for gross national income (GNI) per capita**.

		Psychiatrist density
Women	Pearson correlation	0.508
	Significance (two-tailed)	0.007
	df	25
Men	Pearson correlation	0.492
	Significance (two-tailed)	0.009
	df	25

## Discussion

### Suicide Rates and Psychiatrist Density

This study suggests that higher suicide rates are associated with higher number of psychiatrists working in mental health per 100,000 people. This observation is consistent with previous reports, suggesting that the PD positively correlates with suicide rates (11, 22, 23). The most likely explanation for this association is that the higher suicide rates directly and/or indirectly affect the decisions made by policy- and lawmakers regarding mental health services and how many psychiatrists need to be trained. Indeed, there is very considerable evidence that suicide rates may affect national policies related to mental health and suicide prevention (24–28). Also, the registration of suicide cases may be better in countries with a higher PD and higher income (23). This may contribute to the observed association between suicide rates and PD. Additionally, there are the differences between countries with regard to suicide prevention and other mental health services related to the differences in psychiatric education and training (29). This factor may also play a role in the observed associations.

### GNI and Suicide Rates in Men

The presence of a trend toward a negative correlation between the GNI and suicide rates in men is consistent with observations that the economic factors, such as income, wealth, and employment status, may affect suicidality in men (13–15, 30, 31). For example, I have previously observed that per capita income is related to suicide rates in men but not in women: men in countries with lower per capita income commit suicide more frequently than men in countries with higher per capita income (15). Another example is a British study which found that each 10% increase in the number of unemployed men was significantly associated with a 1.4% increase in male suicides (31).

### Psychiatrist Density and GNI

This study shows that there is a higher PD in countries with the higher GNI per capita. This is an expected finding: countries with more resources have more physicians, including psychiatrists (11, 22, 23, 32). Median mental health expenditures per capita around the world are $1.63 with large variation among income groups, ranging from $0.20 in low-income countries to $44.84 in high-income countries (32). There is a strong positive correlation between mental health spending per capita and the GNI per capita (32).

### Limitations

The results of this study should be treated with caution because many confounding variables, such as cultural and religious differences between countries have not been taken into account. Also, the quality of training of psychiatrists is probably different in different countries. It is also important to note that the income in the European Union countries is higher than in many other countries. Therefore, the results of this study may not be generalizable to low-income countries.

## Conclusion

Both mental health services and socioeconomic improvements are needed to reduce suicide rates. Probably, psychiatric services prevent many suicides. Absence of suicide produces no data. It is difficult to notice successful suicide prevention. Future epidemiological, ecological, psychological, and neurobiological studies of suicidality are merited and may lead to the development of treatments which will reduce suicide rates.

## Author Contributions

LS has designed the study, conducted literature searches and statistical analysis, and wrote the manuscript.

## Conflict of Interest Statement

The author declares that the research was conducted in the absence of any commercial or financial relationships that could be construed as a potential conflict of interest.
